# Deciphering the epigenetic role of long non‐coding RNAs in mood disorders: Focus on human brain studies

**DOI:** 10.1002/ctm2.70135

**Published:** 2025-03-04

**Authors:** Bhaskar Roy, Anuj K. Verma, Yu Funahashi, Yogesh Dwivedi

**Affiliations:** ^1^ Department of Psychiatry and Behavioral Neurobiology University of Alabama at Birmingham Birmingham Alabama USA; ^2^ Department of Neuropsychiatry, Molecules and Function Ehime University Graduate School of Medicine Toon Ehime Japan

**Keywords:** bipolar disorder, epigenetics, gene regulation, human postmortem brain, long non‐coding RNA, major depressive disorder

## Abstract

Epigenetics plays a central role in neuropsychiatric disorders, contributing significantly to their complexity and manifestation. Major depressive disorder (MDD) and bipolar disorder (BD) have profound impact on mood, affect and cognition. Emerging evidence suggests that epigenetic modification of genes plays a pivotal role in the pathogenesis of both MDD and BD. Long non‐coding RNAs (lncRNA) constitute a heterogeneous class of transcripts and have emerged as crucial regulators of epigenetic processes, offering promising insights into the pathophysiology of various diseases. Despite their limited coding potential, lncRNAs are known to play a critical role in achieving global transcriptomic regulation in a spatiotemporal fashion, especially in complex tissue like the brain. This review aims to discuss the specific dysregulation of lncRNAs so far observed in the brains of MDD and BD patients and understand their mechanistic contributions to the disease pathogenesis.

**Key points:**

- Brain‐centric lncRNAs regulate gene networks, and their disruption is linked to MDD.
- In MDD, altered lncRNAs disrupt gene regulation by changing chromatin looping or modifying chromatin accessibility.
- These changes lead to neuronal dysfunction, affecting neural circuitry and synaptic plasticity.
- The result is impaired brain function, contributing to the symptoms of MDD.

## Graphical abstract

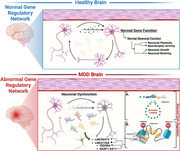

## 1 INTRODUCTION

Major depressive disorder (MDD) and bipolar disorder (BD) both pose significant public health challenges, affecting millions of individuals worldwide. According to the DSM‐V, these two most common mood disorders are represented by fluctuating moods and have been related to multi‐faceted dysfunctionality across emotional, cognitive and behavioural domains.^1^ These disorders affect individuals across their lifespans, with notable prevalence rates in the United States. Recently, it has been shown that approximately 21.4% of US adults experience a mood disorder at some point in their lives, with higher rates among females and adolescents.^2^, ^3^ However, staggering impacts have been seen globally due to the differing prevalence rates of BD and MDD. Amongst them, BD, as one of the most prevalent psychiatric disorders, affects 1–4% of the population.^4^ Conversely, MDD, which is equally debilitating, afflicts up to 12% of adults globally, with a higher prevalence in young adults, women and older people.^5^, ^6^ Both disorders carry an elevated risk of suicide, with an estimated 31% of MDD cases and 34% of BD subjects attempting suicide at least once in their lifetime.^7^, ^8^

The aetiology of mood disorders depends on several factors, including genetic, environmental and psychological; however, vulnerability to stress is the key to their development.^9^, ^10^ Recent molecular studies in the brain have highlighted altered gene expression dynamics as central to the pathogenesis of MDD and BD.^11^, ^12^ Now, it is gaining interest that tress‐associated environmental influences contribute to transcriptomic perturbations in the brains of affected individuals through epigenetic mechanisms, further complicating our understanding of what we learned earlier.^13^ Despite all these, the underlying epigenetic changes and the associated maladaptivity in MDD and BD brains so far mostly remain elusive to us, with uphill challenges to elucidate their roles in gene regulation within the diseased brain. Long non‐coding RNAs (lncRNAs) have emerged as crucial epigenetic regulators, offering potential insights into the pathophysiology of mood disorders.^14^ Lately, it has been shown that lncRNAs are outstanding in bridging the gaps between gene expression changes and cellular processes by creating a complex regulatory landscape. In this connection, the extraordinary ability of lncRNAs to tether intricate gene functions may offer promising avenues for exploring the pathophysiology of mood disorders.^15^, ^16^ So far, converging reports following clinical and preclinical studies have provided valuable insights regarding the functional roles of lncRNAs in mood‐related behaviours and neurobiological processes. However, further research is needed to unravel the complex regulatory roles of lncRNAs and their implications for mood disorder diagnosis, prognosis and treatment.

In the brain, lncRNAs constitute a heterogeneous class of transcripts, contributing significantly to the expressed transcriptome.^17^ Despite their limited coding potential and low expression levels, lncRNAs play critical roles in spatio‐temporal regulation of transcriptomic.^18^, ^19^, ^20^ Their structural features distinguish them from protein‐coding mRNAs, including 5′‐methyl capping and polyadenylated tails. While their primary sequence conservation may be lower, lncRNAs exhibit discernible functional relationships regulating complex cellular processes. Dysregulated lncRNA expression has been associated with various developmental processes and disease pathogenesis. Functionally, lncRNAs act as epigenetic mediators, modulating information processing pathways through cis and trans regulation.^20^ Their diverse regulatory functions make them potential therapeutic targets for modulating gene function.^21^

This review offers a comprehensive overview of recent studies examining the role of lncRNA in mood disorders, with specific emphasis on findings derived from human postmortem brains. The primary focus of this review is to elucidate the mechanisms through which lncRNAs influence MDD and BD. Until recently, the roles of lncRNA were not very clear to understand the pathophysiology of mood disorders. In MDD brain, increasing knowledge about lncRNAs has started pouring in due to expanding research across preclinical and clinical models. However, at this point, their role in the BD brain is seriously limited due to a lack studies. Therefore, this report discusses the presumptive roles of lncRNAs that might be associated with the pathophysiology of BD and highlights the need for further research in this area.

## 2 METHODS

The primary criteria used to search the circulating miRNA‐associated reports were based on studies highlighting the role of lncRNA in MDD and BD brains. With the help of the PubMed database, the search was performed to primarily include original articles for the last five years using various keywords to increase the chance of article retrieval, falling under the criteria mentioned previously. The following keywords were used for retrieval: ‘Long noncoding RNAs’ OR ‘lncRNA’ AND ‘Major depressive disorder’ OR ‘MDD’ OR ‘Major depression’ AND ‘Bipolar disorder’ OR ‘BD’ AND ‘Human’ OR ‘Postmortem brain’ AND ‘Gene regulation’.

## 3 FEATURES OF lncRNA BIOSYNTHESIS, GENE STRUCTURE AND FUNCTION

**FIGURE 1:**
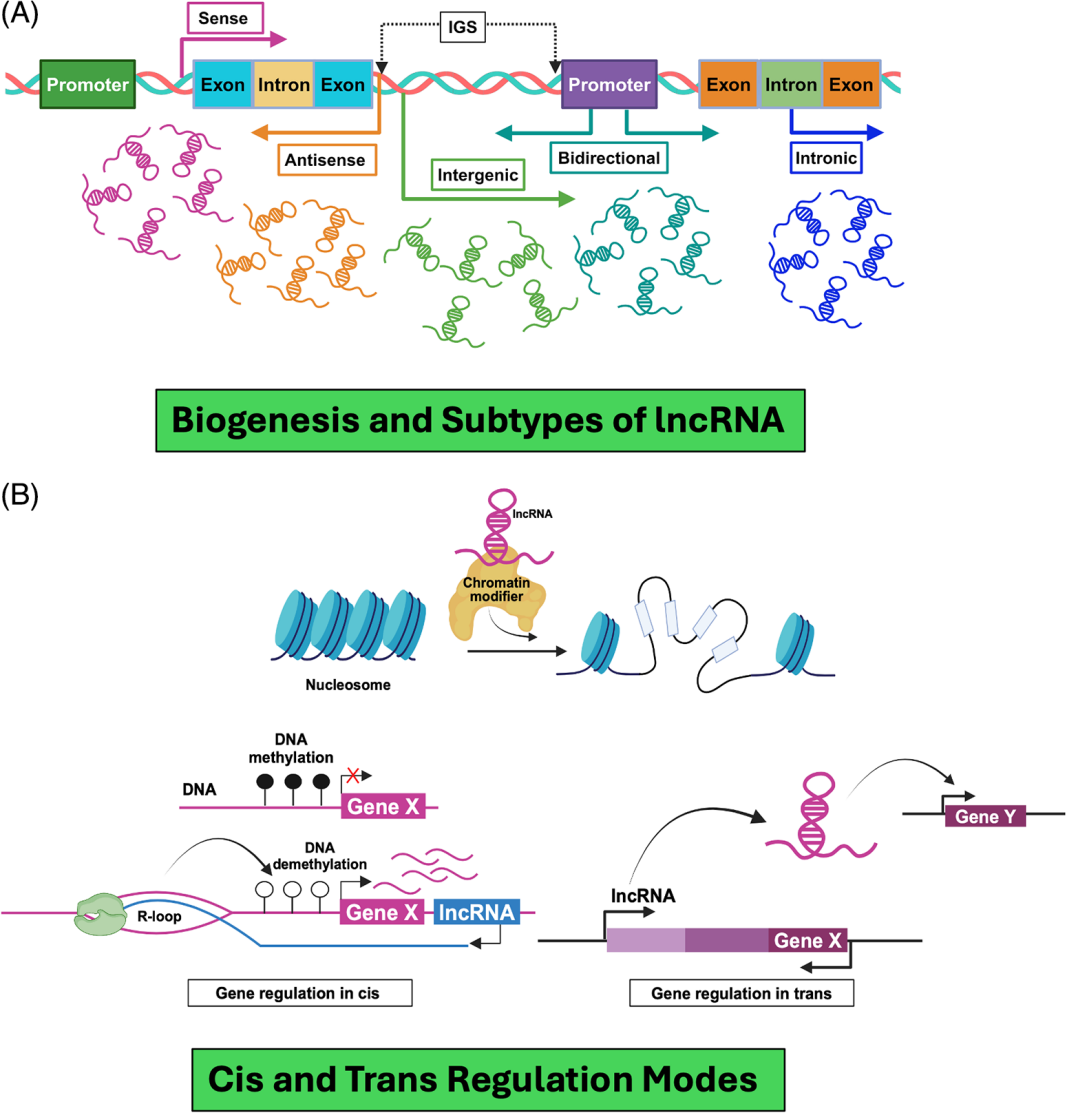
Long non‐coding RNAs (lncRNAs) and their mechanisms of action. (A) lncRNA biotypes. The illustration outlines the classification of lncRNAs based on their genomic location and origin. (B) The diagram illustrates how lncRNAs interact with chromatin modifiers to regulate gene expression. These lncRNAs guide modifiers like DNA methylation enzymes to specific target‐gene promoters, activating or suppressing transcription locally (cis) or at distant sites (trans). Both cis‐acting and trans‐acting lncRNAs modulate the chromatin environment by interacting with DNA directly or indirectly through protein intermediaries, thereby influencing chromatin structure and gene regulation.

Unlike other non‐coding RNAs, biosynthesis of lncRNA is rather complicated, encompassing a complex series of molecular events that ensure proper synthesis, processing, localisation and stability within the cell.^19^ Based on their genomic location and origin, the lncRNAs have been catalogued into different subtypes.^22^ As defined by GENCODE, there are primarily five biotypes (e.g., antisense lncRNAs, sense‐overlapping lncRNAs, sense intronic lncRNAs, long intergenic lncRNAs or lincRNAs and bidirectional lncRNAs) of lncRNAs ^23^ (Figure 1A). Antisense lncRNAs are found on the opposite strand of protein‐coding genes and can overlap with their exons or introns. They are known to regulate the genes they overlap with. Sense‐overlapping lncRNAs have the same orientation as protein‐coding genes and overlap with their introns but not their exons. Sense intronic lncRNAs are located entirely within the introns of protein‐coding genes without touching exons. Long intergenic non‐coding RNAs (lincRNAs) come from regions between protein‐coding genes and are usually located at least 5 kb away from the nearest one. Bidirectional lncRNAs are transcribed from regions within 1 kb of a protein‐coding gene's start site, oriented head‐to‐head with the protein‐coding gene.^24^ Last, the processed transcripts are complex and originate from regions without an open reading frame (ORF), so they do not fit into any other category.^25^

Unlike other non‐coding RNAs, lncRNAs are mostly transcribed from genomic DNA by RNA polymerase II or III inside the nucleus. Most lncRNAs, upon transcription, are capped on the 5′ end with a monomethyl guanosine cap to prevent degradation by enzymes (occurs in eukaryotes). The cap is a guanosine residue with a methyl group attached to a ribose sugar, connected by triphosphate linkages to the 5′ end of the lncRNA. Different types of lncRNAs may be differentiated based on the cap. For example, sno‐lncRNAs can be identified by a snoRNA cap at either the 5′ end of the transcript or the 5′ and 3′ end. There may also be the addition of a poly‐A‐tail to the lncRNA transcript. PolyA synthetase is responsible for this tailing by adding one adenine at a time to the 3′ end of the transcript, varying between 65 and 120 adenines added. In some cases, a poly‐A‐tail can be substituted for a hairpin loop.^20^, ^26^, ^27^, ^28^

LncRNAs are composed of introns and exons, which can be spliced in various ways. The splicing often determines their ultimate function and where they will be localised in the cell. Splicing is done by what is referred to as a spliceosome, a combination of proteins and RNAs whose purpose is to snip the transcript at designated sites. The spliceosome complex identifies the 5′ and the 3′ nick sites and pulls the two sites together to form a loop. The loop can be trimmed and released, leaving the two ends to be put back together by the spliceosome and leaving the linear transcript intact and without the trimmed portion. The trimmed sites vary depending on the function of lncRNAs. Due to the variability in trimming sites, one transcript can acquire many end states and thus may produce many different mature lncRNAs. Some lncRNAs may even be found in circular form (circRNA), and these often originate from the splicing of other RNA transcripts, which leaves a circular lncRNA behind.^19^, ^29^, ^30^

Post‐transcriptional processing of lncRNA is often followed by their association with RNA‐binding proteins and processing factors to form ribonucleoprotein (RNP) complexes. The RNP complexes further facilitate RNA folding, maturation and quality control, that collectively ensure proper cell functioning. Subsequently, mature lncRNAs are transported from the nucleus to the cytoplasm through nuclear pore complexes. Export factors such as Exportin‐1 mediate this nuclear export process, which recognises specific RNA export signals and facilitates the translocation of RNAs across the nuclear envelope. Once in the cytoplasm, lncRNAs may undergo further processing or localisation to specific subcellular compartments. It has been suggested that RNA localisation signals within conserved lncRNA sequences and interactions with RNA‐binding proteins dictate their subcellular localisation patterns. Some lncRNAs may also localise to cytoplasmic granules or RNA processing bodies, where they participate in RNA metabolism and regulatory processes. It has been noted that approximately 70% of the lncRNAs are bound to a polysome in the cytoplasm. The anchorage happens via a specific sequence on the 5′ end of the lncRNA, resembling the 5′ termini of protein‐coding mRNA. This is one of the mechanisms for lncRNA to be localised to polysomes. Likewise, lncRNAs can also be localised to the mitochondria.^22^, ^31^, ^32^

It is pertinent to mention that the biosynthesis of lncRNAs is a complex process but is essential for their diverse functions in gene regulation, sense‐antisense blocking for gene translation, chromatin organisation and orchestrating cellular signalling pathways.^33^ Their intricate involvement spans various levels of gene regulation, including transcriptional, epigenetic, post‐transcriptional and subcellular processes.^34^ As part of the gene regulation process, lncRNAs frequently interact with chromatin modifiers (e.g., DNA methylation) and guide them to specific target‐gene promoters.^35^ This recruitment facilitates either the activation or suppression of transcription, locally in a cis or distantly in a trans manner, often targeting multiple loci simultaneously.^18^ Both cis‐acting and trans‐acting nuclear lncRNAs exert their effects mechanistically by establishing interactions with DNA to modulate the chromatin environment.^36^, ^37^ This chromatin modulation can occur indirectly through a tripartite interaction between RNA, DNA and proteins, thereby influencing the three‐dimensional (3D) chromatin structure (Figure 1B).

## 4 GENE REGULATORY FUNCTIONS OF lncRNAs

**FIGURE 2:**
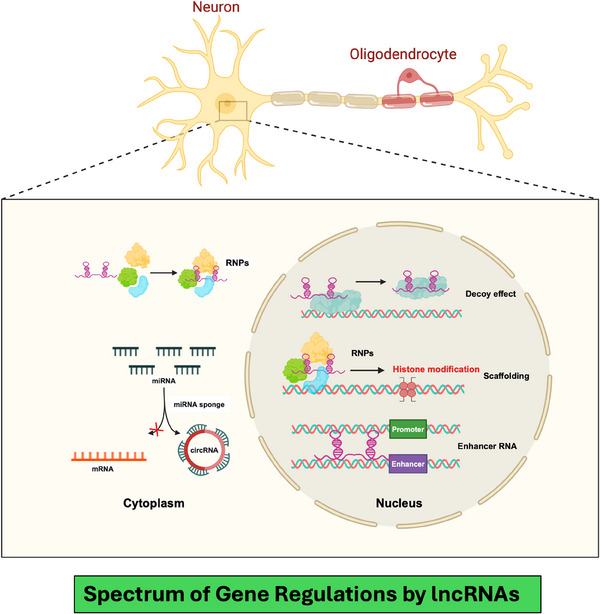
The diagram highlights the multifaceted roles of long non‐coding RNAs (lncRNAs) in gene expression regulation within the neuronal environment. lncRNAs modulate enhancer activity, chromatin looping and epigenetic modifications to regulate transcription. Post‐transcriptionally, they affect alternative splicing, mRNA localisation and cytoplasmic granule assembly. Additionally, some lncRNAs contribute to nuclear body formation. Acting as scaffolds or decoys, lncRNAs assemble ribonucleoprotein complexes or competitively bind regulatory molecules, demonstrating their dynamic interplay with the neuronal environment and their impact on gene expression in both neural health and disease.

It has been well established that lncRNAs can influence various cellular processes due to their ability to regulate global gene expression. As explained in Figure 2, they function in transcriptional regulation by modulating enhancer activity, chromatin looping and epigenetic modifications.^38^, ^39^ Post‐transcriptionally, lncRNAs influence alternative splicing, mRNA localisation and cytoplasmic granule assembly. Some lncRNAs contribute to nuclear body formation.^40^ As scaffolds or decoys, lncRNAs assemble RNP complexes or competitively bind regulatory molecules.^19^ These mechanisms highlight the dynamic interplay between lncRNAs and the cellular environment, shaping gene expression in development and disease.

As mentioned in the preceding section, lncRNAs play a central role in higher‐order gene regulation.^19^, ^41^ During evolution, they have achieved an extraordinary fleet organising the chromatin structure and arranging the histone octamers, two essential epigenetic mechanisms critical in gene expression and neuronal function.^42^ One mechanism by which lncRNAs influence neuronal functions is through the formation and modulation of chromatin loops, which bring specific enhancer and promoter regions in contact, thereby controlling the transcription of genes.^43^ For example, the lncRNA NEAT1 interacts with chromatin architectural proteins, such as CTCF and cohesin, facilitating chromatin loop formation that activates gene networks necessary for synaptogenesis and neuronal maturation.^44^ By recruiting chromatin‐modifying enzymes, NEAT1 also regulates histone acetylation and methylation at target loci, further fine‐tuning gene expression in synapse formation and stability.^45^, ^46^ Additionally, lncRNAs such as MALAT1 contribute to neurogenesis and synaptic plasticity by guiding histone modification complexes to promoters of genes involved in neuronal differentiation and synaptic function.^44^, ^47^ MALAT1 interacts with histone methyltransferases like EZH2, part of the Polycomb Repressive Complex 2, to promote repressive histone marks at specific loci, temporally controlling gene silencing and activation crucial for neural circuit formation and plasticity.^48^, ^49^, ^50^ These processes help maintain synaptic strength and adaptability in response to changing environmental stimuli, underlying learning and memory formation.^46^ Disruption in these lncRNA‐mediated chromatin and histone modification pathways has been linked to neurodevelopmental and neuropsychiatric disorders.^51^ Together, they suggest that aberrant lncRNA activity may result in dysregulated neuronal circuits and impaired cognitive function. Although the fundamental mechanisms underscored their role in maintaining essential neuronal functions, however, more studies are needed to explore the same in MDD and BD brains. In summary, lncRNAs influence the chromatin landscape in a highly specific manner by orchestrating chromatin loops and histone modifications for precise gene regulation. Thus, dysregulation of lncRNA‐mediated chromatin and histone dynamics can impact the core processes of neurogenesis, synaptogenesis and synaptic plasticity, potentially contributing to neurodevelopmental and neuropsychiatric disabilities in the human brain.^52^ The following section further highlights some critical gene regulatory functions mediated by lncRNAs.^53^

### 4.1 Transcriptional regulation

#### 4.1.1 Enhancer or repressor function

lncRNAs act as efficient transcription regulators by binding to enhancer regions and either facilitating the assembly of transcriptional activators or repressors. This, in turn, influences the recruitment of RNA polymerase II to target promoters. This interaction can activate or suppress context‐dependent gene expression, exerting precise control over gene regulatory networks.^54^

#### 4.1.2 Chromatin looping

Through participation in chromatin looping events, a specialised class of lncRNAs, or enhancer RNAs (eRNA), bring distal enhancer elements into proximity with target gene promoters.^55^, ^56^, ^57^ This spatial organisation accomplished by this special class of lncRNAs enhances the interaction between regulatory elements and the transcriptional machinery.^58^, ^59^ The end product is dynamic gene expression regulation in response to endogenous developmental cues or exogenous environmental stimuli under normal and abnormal conditions.^60^

#### 4.1.3 Transcriptional interference

Certain lncRNAs interfere with the transcription of adjacent genes through transcriptional collision or recruitment of chromatin‐modifying complexes on their neighbouring sites. With that lncRNAs can influence the cellular phenotype by modulating the expression levels of neighbouring genes. This highlights their intricate regulatory roles in gene expression dynamics.^19^

### 4.2 Epigenetic regulation

#### 4.2.1 Histone modification dynamics

lncRNAs play a pivotal role in modulating the dynamics of histone modifications, thereby influencing chromatin accessibility and gene expression. Acting as guides for histone modifiers, lncRNAs target specific genomic loci, regulating the deposition or removal of histone marks associated with active or repressive chromatin states. This epigenetic regulation fine‐tunes gene expression programs in response to cellular signals or environmental cues, contributing to cellular differentiation and development.^36^

#### 4.2.2 DNA methylation patterns

Some lncRNAs are intricately involved in DNA methylation processes by recruiting DNA methyltransferases or demethylases to target genomic regions. By regulating DNA methylation patterns, these lncRNAs can significantly impact gene expression programs and cellular differentiation processes, influencing cellular identity and function.^61^

### 4.3 Post‐transcriptional regulation

#### 4.3.1 Alternative splicing regulation

Specific lncRNAs modulate alternative splicing events through interactions with splicing factors or by guiding spliceosome assembly. This regulatory mechanism produces splice variants with distinct functional properties, impacting cellular phenotypes and responses. By regulating the diversity of the transcriptome, lncRNAs contribute to the complexity of gene regulatory networks and cellular processes.^34^, ^62^

#### 4.3.2 mRNA localisation and transport

Cytoplasmic lncRNAs interact with mRNA molecules and regulate their subcellular localisation and transport dynamics. By influencing mRNA localisation, these lncRNAs play crucial roles in regulating localised protein synthesis and cellular signalling processes within specific subcellular compartments. This spatial regulation of mRNA localisation ensures precise control over gene expression in response to cellular cues or environmental stimuli.^63^

### 4.4 Subcellular localisation

#### 4.4.1 Nuclear body formation

Certain lncRNAs contribute to forming nuclear bodies, such as paraspeckles or nuclear speckles, acting as structural components or molecular scaffolds. These nuclear bodies serve as RNA processing, storage or sequestration sites, thereby regulating gene expression and cellular responses. By organising nuclear architecture, lncRNAs contribute to the spatial regulation of gene expression and nuclear processes.^64^

#### 4.4.2 Cytoplasmic granule assembly

Cytoplasmic lncRNAs often act as cellular rheostats that interact with RNA‐binding proteins and other granule components to assemble RNA granules, such as stress granules or P‐bodies. These RNA granules are dynamic hubs for mRNA metabolism, translation regulation and quality control mechanisms. By regulating RNA granule assembly, lncRNAs influence cellular stress responses, protein synthesis and mRNA turnover, contributing to cellular homeostasis and adaptation.^65^, ^66^

### 4.5 Scaffold and decoy functions

#### 4.5.1 RNP complex assembly

lncRNAs act as scaffolds for assembling RNP complexes, bringing together multiple RNA‐binding proteins and regulatory factors. These RNP complexes modulate various aspects of RNA metabolism, including splicing, editing and localisation. By facilitating RNP complex assembly, lncRNAs regulate RNA processing and localisation dynamics, contributing to the diversity and complexity of gene expression programs.^67^, ^68^

#### 4.5.2 Competitive binding as decoys

Some lncRNAs function as competitive inhibitors or decoys by sequestering regulatory molecules away from their target sites. By competing for binding with miRNAs, RNA‐binding proteins or transcription factors, these lncRNAs modulate the activity of regulatory networks and influence gene expression patterns. This competitive binding regulates gene expression in response to cellular signals or environmental cues, allowing cells to adapt to changing conditions and maintain homeostasis.^34^, ^69^

Through the above‐described mechanisms, lncRNAs tune gene expression programs, cellular processes and signalling pathways, contributing to the complexity and versatility of biological processes. Their intricate regulatory roles highlight the dynamic interplay between lncRNAs and the cellular microenvironment upon receiving cues from external influences and shaping gene expression dynamics and cellular responses in health and development.^70^ Although a substantial number of studies have been conducted to decipher the role of lncRNAs on gene regulation, understanding the core mechanism causing MDD and BD pathophysiology is still in infancy. It has been anticipated that any of the events may turn pathogenic under cellular maladaptivity and could be the molecular core of mood disorders.

## 5 BRAIN‐CENTRIC lncRScape OF MOOD DISORDER

Once relegated to the sidelines of genomic research, lncRNAs have emerged as pivotal players in explaining many previously unexplained disconnects in MDD and BD brains beyond traditional protein‐coding gene functions. Recently, groundbreaking studies have illuminated the involvement of lncRNAs in stress‐related mood dysfunctionalities, offering novel insights into the pathophysiology of these complex conditions.^71^, ^72^, ^73^, ^74^ Here, we synthesise and elucidate the roles of lncRNAs in brain functions primarily associated with MDD and BD. Whereas several studies point to the role of lncRNAs in major depression, our literature search found a significant knowledge gap to underscore the mechanistic roles of lncRNAs studied in the context of BD. So far, to our knowledge, no brain‐centric lncRNA study has been reported focusing on the BD (Table 1).

**TABLE 1:** Long non‐coding RNA (LncRNA) expression changes in brains of MDD and suicide subjects.

Disorder	Brain area studied	lncRNA expression changes	Findings	References
MDD‐suicide	Rostral anterior cingulate cortex	Antisense (CTC‐487M23.5, RP11‐273G15.2, RP11‐326I11.3 and RP1‐269M15.3), sense overlapping (RP11‐96D1.10), sense‐intronic (CTD‐2647L4.4), intergenic (RP11‐453F18_B.1, RP11‐434C1.1 and ZNF833P)	Differentially expressed	^75^
MDD	Ventromedial prefrontal cortex, dorsolateral prefrontal cortex, orbitofrontal cortex, nucleus accumbens, ventral subiculum	LINC00473	Sex‐specific expression downregulation	^76^
MDD	Rostral anterior cingulate cortex	FEDORA (RP11‐298D21.1)	Sex‐specific expression upregulation	^81^
Suicide	Prefrontal cortex	LINC01268	Expression upregulation	^82^

The pathophysiological mechanisms associated with psychiatric disorders, particularly mood disorders, continue to challenge researchers. The focus has recently expanded beyond protein‐coding genes to explore the role of lncRNAs in these complex disorders. One study employed RNA‐sequencing in a specific brain region, the rostral anterior cingulate cortex, to shed light on the potential involvement of lncRNAs in depression and suicide. The study, encompassing 26 depressed individuals who died by suicide and 24 matched controls, provided a comprehensive analysis of lncRNA expression patterns. Through differential expression analysis, researchers identified 23 lncRNAs showing significant alterations in expression levels (e.g., SNORD3C, RP11‐453F18_B.1, LLNLF‐65H9.1, RP11‐96D.1.10 and RP1‐269M15.3). The study further explored an interplay between lncRNAs and protein‐coding genes, uncovering overlapping and antisense relationships. The finding suggests an association between lncRNA‐mediated gene modifications and interferon signalling, a pivotal component of the innate immune response, underscoring a potential avenue for therapeutic intervention. Furthermore, the study employed weighted gene co‐expression network analysis (WGCNA) to unravel complex networks of co‐expressed genes linked to depression and suicide. This approach unveiled modules of highly interconnected genes, wherein protein‐coding genes associated with differentially expressed lncRNAs played a prominent role. Notably, these protein‐coding genes were not merely proximal but distally located from the associated lncRNAs, suggesting trans‐regulatory mechanisms governing gene expression. The enriched Gene Ontology (GO) terms within these significant modules provided valuable insights into the molecular underpinnings of depression and suicide. Critical biological processes such as cytoskeleton organisation, plasma membrane dynamics, cell adhesion and nuclear functions emerged as potential targets for further investigation. Moreover, the regulation of dendrite development and morphology, crucial for synaptic plasticity and neuronal connectivity, underscored the neurobiological complexity underlying these psychiatric conditions. Overall, this study highlights the importance of lncRNAs as potential regulators of critical molecular functions and biological processes implicated in depression and suicide.^75^

As is well known, depression the prevalence of MDD is higher in females than males, with females being affected at twice the rate. A recent study examined the involvement of sex‐specific changes in lncRNAs in MDD subjects and found a distinct pattern of regulation, not only varying across brain regions but displaying striking sex‐specific alterations. Among various lncRNAs scrutinised, LINC00473 emerged as a primate‐specific gene enriched in neurons, which showed downregulated expression specifically in the prefrontal cortex (PFC) of depressed females, with no such alteration in males. To elucidate the functional significance of LINC00473 in depression, researchers further employed a viral‐mediated gene transfer approach to express LINC00473 in adult mouse PFC neurons. This manipulation emulated the human sex‐specific phenotype, inducing resilience to stress exclusively in female mice. Changes in synaptic function and gene expression accompanied the sex‐specific nature of this resilience. Further insights from studies utilising human neuron‐like cells implicate LINC00473 as a downstream effector of CREB, a crucial transcription factor involved in stress response and neuronal plasticity. CREB signalling is important to play role neuronal plasticity, survival and resilience.^76^ LINC00473 is shown to respond to CREB signalling. Reduced levels of LINC00473 disrupt this CREB signalling pathway, compromising neuronal resilience.^77^ This compromising resilience happens through diminishing levels of CREB and ultimately influences neuroplasticity and stress axis in the brain.^78^ Additionally, LINC00473 affects synaptic plasticity within the PFC.^79^ As previously mentioned, in a small rodent model of female mice, LINC00473 expression decreased the frequency and amplitude of excitatory postsynaptic currents in pyramidal neurons, which may act to stabilise neuronal signalling during stress.^77^, ^80^ This dampening of excitatory synaptic activity aligns with improved stress resilience, as excessive excitatory signalling is associated with heightened stress responses and vulnerability to mood disorders.^79^ Moreover, LINC00473 regulates the expression of genes involved in neurogenesis and inflammation, pathways often implicated in the aetiology of depression.^79^ The study showed that in the PFC, LINC00473 expression significantly reduced the number of stress‐induced transcriptional changes, suggesting its role in maintaining stable gene expression profiles under stress.^79^ This mechanistic understanding offers valuable insights into the molecular pathways underlying stress resilience and vulnerability in depression, particularly in the context of gender‐specific differences in lncRNA responses. In summary, this study identifies LINC00473 as a female‐specific driver of stress resilience aberrant in female depression. By unravelling the interplay between lncRNAs, synaptic function and sex‐specific vulnerability to depression, it opens new avenues for targeted therapeutic interventions and personalised approaches to tackling this debilitating disorder. Further research is needed to fully decipher the complex regulatory networks orchestrated by lncRNAs and their implications for gender‐specific psychiatric disorders.^79^

Another recent study explored the molecular landscape of the human brain, focusing on the role of lncRNAs to shed light on the gender disparities observed in depression. By analysing postmortem human brain tissue, researchers uncovered significant baseline differences in the expression of lncRNAs between males and females in the context of depression. One noteworthy lncRNA emerged among the numerous lncRNAs scrutinised, that is, RP11‐298D21.1, aptly termed FEDORA. According to the study, this lncRNA was enriched in both oligodendrocytes and neurons and showed significant expression upregulation, specifically in the PFC of depressed females. To elucidate the functional implications of FEDORA dysregulation, researchers employed viral vector‐mediated expression of FEDORA selectively in either neurons or oligodendrocytes within the PFC of mice. Strikingly, this manipulation induced depression‐like behavioural abnormalities exclusively in female mice, underscoring the sex‐specific nature of FEDORA and its potential impact on brain function and behaviour. These changes were associated with cell type‐specific alterations in synaptic properties, myelin thickness and gene expression, highlighting the multifaceted role of lncRNAs in shaping neural circuits and behaviour. Moreover, the clinical relevance of FEDORA extends beyond the realm of experimental models, as evidenced by its diagnostic implications for depressed women. Notably, blood levels of FEDORA emerged as potential biomarkers, offering valuable insights into the diagnosis and treatment response of depression, particularly in women. Furthermore, the association between FEDORA levels and clinical response to ketamine treatment underscored its potential as a prognostic indicator and therapeutic target in depression management. In essence, this study unveils the pivotal role played by lncRNAs, particularly FEDORA, in carving out the sex‐specific landscape of the brain and contributing to the gender differences observed in depression.^81^

A recent study explored the role of lincRNAs LINC01268 to understand the interplay between genetics, brain function and behaviour in the context of aggressive behaviour and suicide. Employing a multi‐pronged approach, the authors analysed RNA sequencing data from human brain tissue to corroborate the prior postmortem association of LINC01268 with suicide by violent means. Consistent with previous findings, they observed significantly higher expression of LINC01268 in the PFC of individuals who died by violent suicide compared to non‐suicides and suicides by non‐violent means. Extending their investigation to the behavioural level in clinical samples, the authors leveraged a genetic variant associated with LINC01268 expression to elucidate its impact on in vivo prefrontal physiology related to behavioural control. Carriers of the minor allele of this single nucleoptide polymorphism (SNP), linked to increased LINC01268 expression in the brain, exhibited higher scores on a lifetime aggression questionnaire and showed diminished engagement of the PFC during functional magnetic resonance imaging (fMRI) tasks involving the processing of angry faces. This finding provides compelling evidence linking LINC01268 expression with emotional regulation and aggressive tendencies in human subjects. Furthermore, WGCNA highlighted the underlying biological processes associated with the LINC01268 co‐expression network, showing the involvement of the immune responses. This suggests a potential mechanistic link between LINC01268 and immune‐related genes, implying a relationship between neuroinflammation and emotional dysregulation in the context of aggressive behaviour and suicide. In conclusion, this study offers insights into the role of LINC01268 in shaping emotional regulation, aggressive behaviour and suicide by violent means. By elucidating the biological dynamics underlying these complex phenomena, including the modulation of immune‐related genes, it opens new avenues for understanding and potentially intervening in the pathophysiology of aggressive behaviour and suicide. However, further research is warranted to unravel the precise mechanisms by which LINC01268 exerts its influence and to explore its therapeutic potential in mitigating the risk of violent suicidal behaviour.^82^

The pathophysiology of MDD, particularly in response to chronic stress, can lead to alterations in gene expression and regulatory molecules. The hippocampus, a brain region sensitive to stress, holds clinical significance in understanding the outcomes of antidepressant response in MDD patients. In a study, magnetic resonance imaging (MRI) was utilised to measure hippocampal volumes in individuals with MDD (*N* = 201) and healthy controls (HC = 104). Simultaneously, RNA sequencing and DNA methylation (Infinium MethylationEpic Beadchip) were employed to evaluate gene expression and methylation levels, respectively. The investigation explored the association between hippocampal volume, RNA expression and DNA methylation. Sixty RNAs were identified as differentially expressed between MDD and control groups, with 21 exhibiting differential methylation and seven showing a correlation between methylation and expression. Notably, a negative association was observed between the expression of Brain Abundant Membrane Attached Signal Protein 1 antisense 1 RNA (BASP1‐AS1) and right hippocampal tail volume in the MDD group. Furthermore, the duration of the current depressive episode moderated this association, suggesting a dynamic relationship between BASP1‐AS1 expression, hippocampal volume and depressive symptomatology. These findings shed light on the molecular mechanisms underlying hippocampal alterations in MDD and highlight the potential role of BASP1‐AS1 as a biomarker or therapeutic target for the disorder.^83^

**FIGURE 3:**
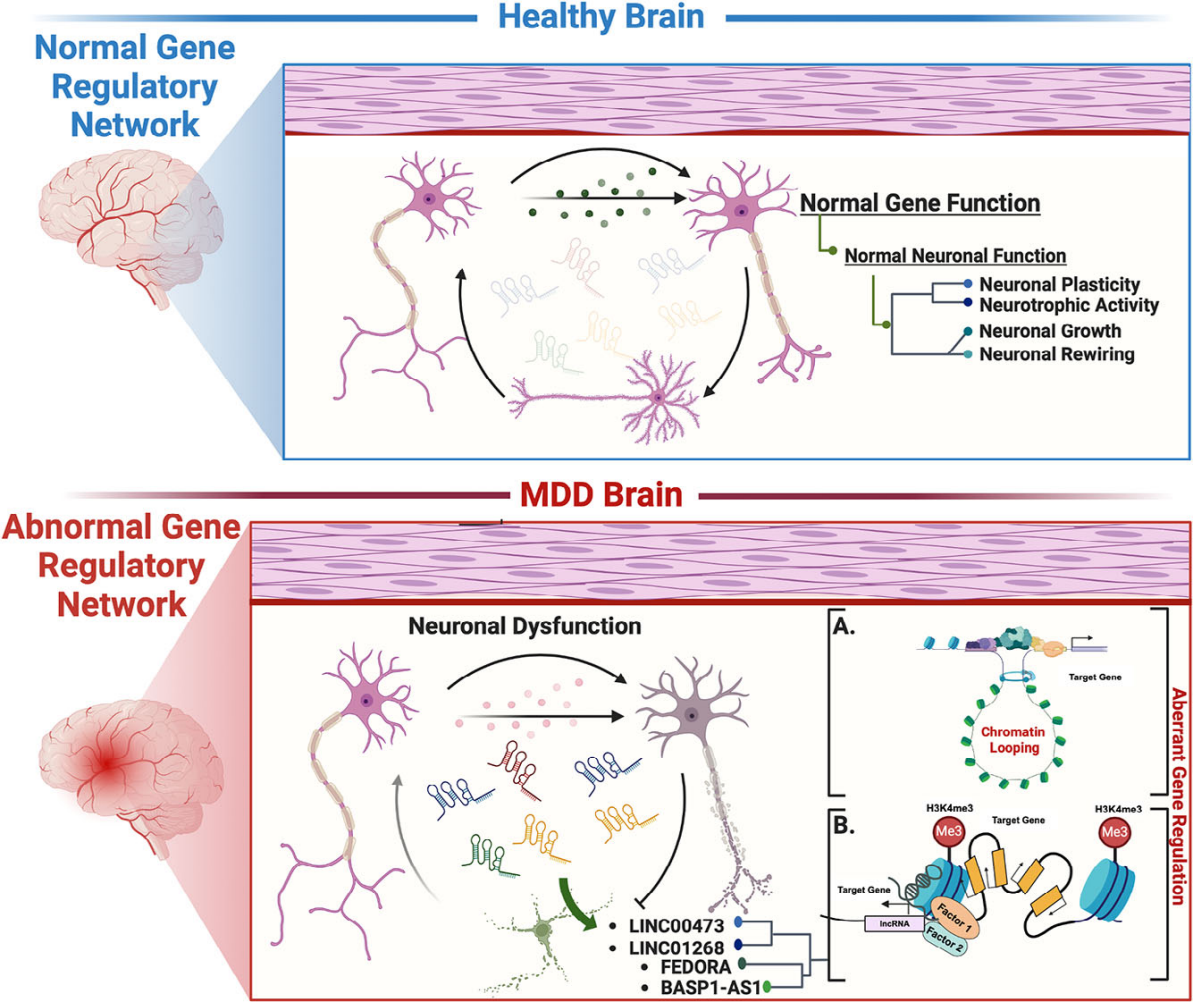
Thematic diagram highlighting the role of brain‐centric lncRNAs in developing MDD pathophysiology. The diagram illustrates how MDD‐associated changes in lncRNAs disrupt normal gene regulatory networks, leading to widespread maladaptivity via: (A) modulating long‐distance chromatin looping or (B) modifying the chromatin accessibility following histone modifications. These changes contrast with the normal regulatory functions in a healthy brain and result in neuronal dysfunctionality, affecting neural circuitry, synaptic plasticity and overall gene regulatory functions.

The above‐discussed reports led us to conclude that elucidating lncRNA‐mediated gene regulatory networks in the context of MDD represents a paradigm shift in psychiatric research. From the interaction between lncRNAs and protein‐coding genes to the sex‐specific alterations observed in MDD, these studies showed the multifaceted roles of lncRNAs in shaping neural circuits, synaptic function and behaviour. Moreover, identifying lncRNAs such as LINC00473 and FEDORA as potential biomarkers and therapeutic targets underscores their clinical relevance and diagnostic implications. However, while these findings offer promising avenues for personalised interventions and targeted therapies, further research is imperative to decipher the precise mechanisms underlying lncRNA‐mediated regulation and its implications in mood disorders. A thematic diagram highlights the role of brain‐centric lncRNAs discussed in developing MDD pathophysiology. This diagram encapsulates the interactions and regulatory functions of lncRNAs, emphasising their significance in neural circuitry, synaptic plasticity and overall gene regulatory functions in MDD (Figure 3).

### 5.1 Exploring potential roles of lncRNAs in BD

As mentioned above, lncRNAs have emerged as crucial regulators of gene expression, with significant implications for neuropsychiatric disorders. Empirical evidence on the role of lncRNAs in BD remains sparse except for one report of circRNA in the medial PFC of BD subjects. In that report, two circRNAs from the NEBL and EPHA3 loci showed increased expression in BD.^84^ EPHA3 is particularly noteworthy because it plays a role in developmental processes within the central nervous system (CNS). Eph receptors, part of the protein‐tyrosine kinase family, and their ligands, ephrins, are crucial for various CNS functions, including neurotransmitter release, glutamate receptor activity and dendritic spine formation.^85^, ^86^ These processes are essential for memory and anxiety, commonly disrupted in BD.^87^ Eventually, Ephs and ephrins could serve as potential targets for pharmacological intervention. Existing research suggests that lncRNAs could contribute to the pathophysiology of this disorder in several other ways. This assumption is primarily based on the data obtained from the peripheral tissues of bipolar patients. For example, in a recent study, next‐generation sequencing has been employed to analyse peripheral lncRNA expression in eight bipolar patients and eight healthy controls. Of 27,348 lncRNAs identified in both groups, 10 showed significant expression differences (false discovery rate < 0.05). Among these, six lncRNAs were up‐regulated, and four were down‐regulated in bipolar patients compared to controls.^84^ In addition, many recent studies have shown that lncRNA in peripheral circulation represents a promising frontier for advancing our understanding of the neurobiological mechanisms involved in the pathogenesis of BD. A collection of these lncRNAs has been recently reviewed in the context of BD.^88^

As lncRNAs may modulate the expression of genes involved in neurotransmitter systems, such as dopamine and serotonin, there is a possibility that they could influence the synthesis, release or reuptake of these neurotransmitters, thus affecting mood stability, which may contribute to the episodic nature of BD.^89^ Additionally, inflammatory processes are increasingly recognised in the pathophysiology of BD, and lncRNAs can regulate the expression of pro‐inflammatory cytokines and other inflammatory mediators.^89^ By modulating neuroinflammation, lncRNAs might influence the severity and progression of BD. It has been recently suggested that lncRNAs can influence chromatin stability through epigenetic modifications, such as DNA methylation and histone modification. It has been long known that dynamic changes in chromatin states are critical for gene expression regulation.^90^ Through these mechanisms, lncRNAs might alter the expression of genes associated with BD susceptibility and resilience. While the current understanding of lncRNAs in BD is primarily derived from peripheral tissues, ongoing research on the brain will likely uncover their precise roles in this disorder. In summary, a mechanistic understanding of lncRNA roles in brain dysfunction in BD can provide distinctive molecular and functional signatures that differentiate it from MDD. This knowledge has the potential to enhance diagnostic precision and lead to more effective, personalised treatments, ultimately improving the management of both BD and MDD.

## 6 FUTURE DIRECTIONS IN lncRNA RESEARCH FOR MOOD DISORDERS

lncRNAs hold promise as therapeutic targets beyond their role as biomarkers, particularly in BD and MDD. However, A comprehensive diagnostic network combining neuroimaging, lncRNAs and other psychological markers could use multiple diagnostic layers to understand better the complexity of neuropsychiatric disease processes as seen in mood disorders.^91^ Molecular and genetic biomarkers are key components and should include not only lncRNAs, but also other non‐coding RNAs such as miRNAs, specific genetic variations and protein markers associated with disease states.^92^ In fact, changes in other non‐coding RNAs are promising in both MDD and BD diagnosis.^93^ Neuroimaging techniques, including magnetic resonance imaging (MRI), fMRI and positron emission tomography (PET) scans, also play a central role in the structural and functional assessment of the brain, including imaging techniques.^94^, ^95^ Structural scanning methods help visualise physical brain abnormalities, while functional imaging methods can examine activity patterns and highlight disruptions in brain networks. Additional imaging techniques like diffusion tensor imaging can add further insights by mapping white matter tracts, while electro encephalogram (EEG) or magentoencephalography (MEG) offers real‐time brain activity data.^96^ In addition, peripheral biomarkers from blood and cerebrospinal fluid (CSF) can proxy the brain and reflect molecular changes related to the pathophysiology of mood disorders.^97^ For example, examining blood‐based biomarkers can indicate levels of inflammation, hormones and metabolic activity in the MDD or BD brains.^98^ At the same time, CSF contains brain‐derived proteins that can reflect disease‐specific changes associated with the CNS.^99^ Thus, analysing the metabolic profiles of blood and CSF from diseased patients can provide valuable insights into the metabolic shifts uniquely related to mood disorders.^100^, ^101^ All the approaches discussed above could be complemented by clinical and medical history, which offers essential background on genetic predisposition, family history and any relevant past medical conditions.^102^ Finally, the effectiveness of the nomothetic network, including the role of lncRNA, would be enhanced by predictive statistical modelling.^103^ Recent advancements in machine learning technology based on statistical modelling combined with diverse data sources can help improve the diagnostic accuracy of MDD and BD in mood disorder patients.^104^ More specifically, the complex algorithms behind the predictive statistics can identify subtle patterns related to disease trajectory or treatment responses in mood disorder patients requiring personalised treatments.^105^, ^106^, ^107^ Thus, building an integrative approach, including lncRNAs as part of a broader network, may allow a more holistic understanding of these two complex disease states.

**FIGURE 4:**
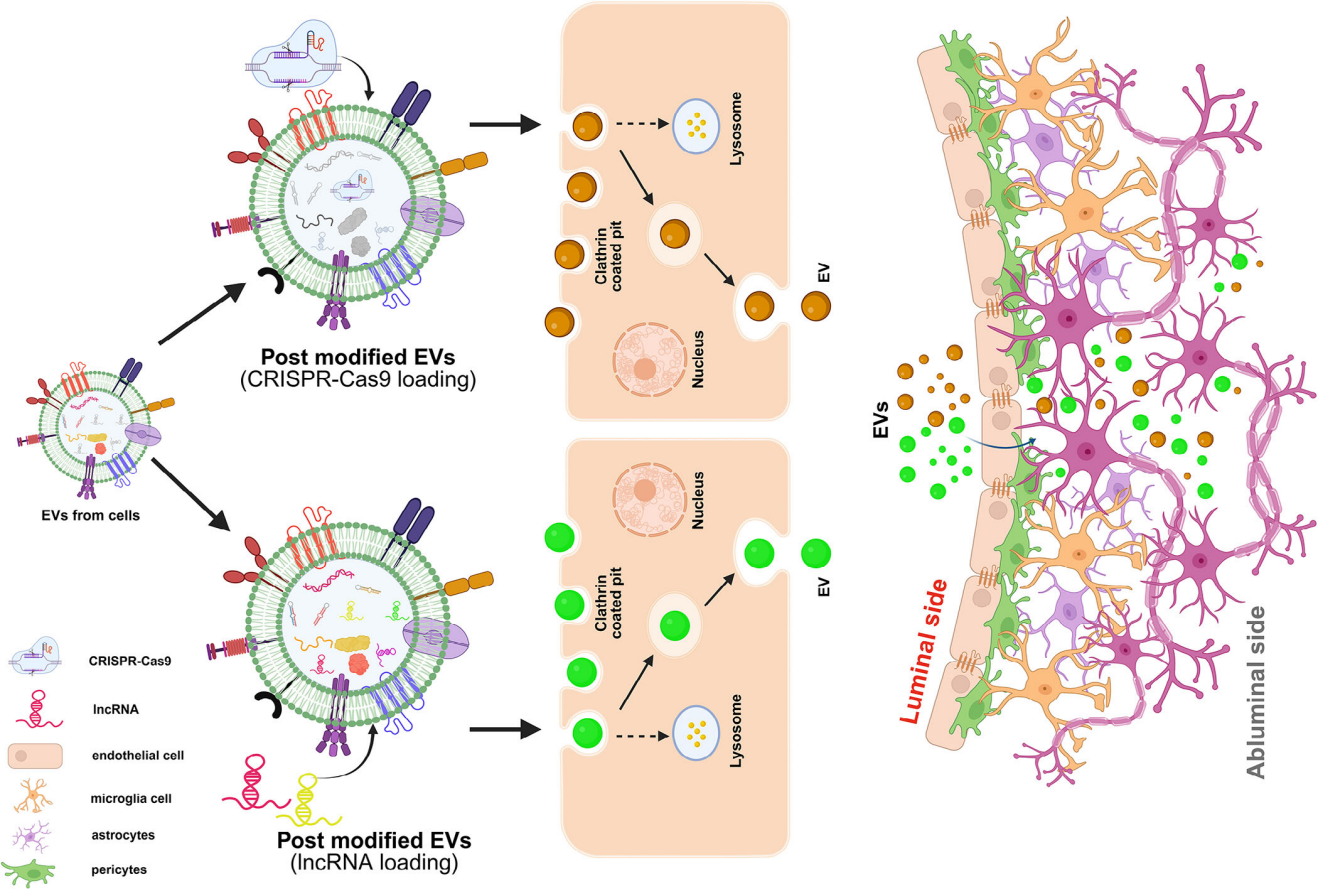
The lncRNA‐mediated targeted therapies (hypothetical) for mood disorders in the brain. The illustration shows future directions with the potential to incorporate extracellular vehicles (EVs) in delivering select lncRNAs poised with genome editing tools like CRISPR–Cas to select brain regions. Conceptually, the engineered EVs with specific surface markers can achieve targeted docking to the selected brain destination by crossing the apparently impermeable blood–brain barrier (BBB).

RNA‐based methods targeting lncRNA transcripts for degradation or interference offer diverse therapeutic avenues. Approaches such as antisense oligonucleotides (ASOs) and small interfering RNAs (siRNAs) are among the most employed therapies approved for clinical use.^108^ ASOs are designed to complement target RNA sequences, disrupt protein translation and induce RNase H cleavage, rendering the transcript non‐functional. In contrast, siRNAs utilise endogenous microRNA machinery to cleave target RNAs or recruit repressive proteins, leading to degradation or inhibition of translation.^109^ Despite the potential, therapies targeting lncRNA molecules have not yet been approved for clinical practice in humans. However, laboratory approaches, including transcriptional inhibition and genome editing following CRISPR–Cas9 and Cas13, hold promise. ASOs, for instance, have shown efficacy in degrading natural antisense transcripts in the CNS in vivo.^108^, ^110^ However, there are some challenges in using the aforementioned tools as they can reduce the therapeutic efficacy due to immunogenic insults that occur during delivery.^111^ They can also show low target specificity and off‐target delivery. Additionally, effective therapy necessitates crossing the blood–brain barrier (BBB), often requiring risky procedures. Emerging methods like a minimally invasive nasal depot offer the potential for safe and effective delivery of therapeutics to the CNS.^112^ Moreover, therapies must exhibit cell subtype specificity and low toxicity. circRNAs are intriguing candidates due to their reduced immunogenicity and potential as miRNA sponges, influencing miRNA function, another class of non‐coding RNA that influences gene expression post‐transcriptionally. Exosomes and other membrane vesicles solve BBB crossing and toxicity concerns. Delivery of glycoprotein‐circSCMH1 via extracellular vesicle intravenous injection has shown promise in animal models.^113^ In Figure 4, we have now illustrated an overview of the various therapeutic directions that have been discussed above. The illustration highlights the potential to incorporate extracellular vehicles (EVs) in delivering select lncRNAs poised with genome editing tools like CRISPR–Cas to select brain regions. Conceptually, the engineered EVs with specific surface markers can achieve targeted docking to the selected brain destination by crossing the apparently impermeable BBB.^114^

A new kind of RNA therapy has been in development, which involves the combination of multiple molecules (DNA, RNA, protein and lipid) that can actively dock on the targeting sites, also called an interactor element or structural element on lncRNA molecules.^115^ This interaction can directly influence the conformation of lncRNA molecules and may potentially alter their functionality. One such example is the interaction of a small molecule NP‐C86 with lncRNA‐UPF1, which impedes the binding of this lncRNA with target protein GAS5, a facilitator of insulin receptors for uptaking into adipocytes.^116^ In RNA therapy, this recent advancement in the multi‐molecule approach represents a promising strategy for targeting lncRNAs.^108^, ^117^ This could be interesting, given the emerging role of lncRNA in understanding mood disorders. This approach leverages the combined effects of various molecules—such as DNA, RNA, proteins and lipids—to specifically modulate gene networks associated with mental health.^118^ The approach has potential applications in targeting stress‐response pathways and other mechanisms underlying neuropsychiatric conditions. One core example is the targeting of lncRNA GAS5, which has been linked to cellular responses to stress and glucocorticoid regulation, with potential implications in depressive disorders.^108^, ^118^ Small molecules designed to interact with GAS5 could alter its binding with glucocorticoid receptors and potentially shift gene expression patterns in a way that modulates stress‐related behaviours.^119^ Additional stabilising agents, such as peptides or proteins, could enhance the molecular structure of the docking interface and increase the specificity of RNA interactions. Lipid‐based nanoparticles are further employed to deliver these molecules effectively across the BBB, addressing a key obstacle in neurological treatments by improving drug permeability and bioavailability.^120^, ^121^ NP‐C86 exemplifies the multi‐molecule approach by targeting the lncRNA UPF1. NP‐C86 may prevent the UPF1‐GAS5 interaction, potentially reducing gene repression and restoring the expression of essential receptors involved in insulin uptake and glucose homeostasis, which could indirectly affect mood regulation.^116^ In neuropsychiatric applications, similar multi‐molecule strategies target other lncRNAs, such as NEAT1 and MALAT1.^74^ NEAT1 has been shown to play a role in neuroinflammatory responses and immune signalling, which are implicated in conditions like depression and schizophrenia.^122^ MALAT1 is associated with synaptic plasticity and cognitive processing, and its modulation could offer therapeutic insights into memory and learning deficits often observed in mood disorders.^49^, ^50^ This multi‐molecule framework addresses the need for specificity in RNA‐based therapies and could reduce the risk of off‐target effects and immune reactions—a significant limitation in many current treatments.^123^ Integrating stabilising agents and delivery systems further supports the therapeutic stability and precision needed for brain‐specific treatments.^124^ However, developing these therapies demands thorough molecular engineering and an advanced understanding of RNA‐protein interactions in the brain, as variations in these pathways may affect treatment outcomes.^125^ Thus, targeted RNA‐based therapy with a multi‐molecule approach has the potential to achieve faster and more durable effects in treating mood disorders than any other conventional methods and could enable clinicians with much‐desired tools to manage patient care more efficiently by driving a new era of precision medicine.

Future research integrating novel sequencing methods and functional characterisations of lncRNAs in the CNS holds the potential to revolutionise therapeutic targeting. Moreover, lncRNAs present a promising avenue for understanding the pathogenic changes in the brain associated with mood disorders, including MDD and BD. By elucidating the intricate regulatory roles of lncRNAs in neural circuits and cellular processes, we can gain deeper insights into the underlying mechanisms of mood disorders, ultimately leading to more effective diagnostic and therapeutic strategies tailored to individual patients. This could involve identifying key lncRNAs involved in the dysregulation of neural pathways implicated in mood disorders, elucidating their interactions with protein‐coding genes and regulatory networks, and validating their diagnostic and therapeutic potential in clinical settings. Additionally, leveraging advanced technologies such as single‐cell sequencing and CRISPR‐based genome editing can further enhance our understanding of the role of lncRNAs in neuropsychiatric illnesses, paving the way for targeted interventions and personalised medicine approaches.

## 7 CONCLUSIONS

In summary, mood disorders like MDD and BD present substantial global health challenges. Recent research has underscored the mechanistic roles of lncRNAs in the pathogenesis of these disorders, revealing significant epigenetic dysregulation. This report has outlined these findings, highlighting the potential of lncRNAs as therapeutic targets. Further research is needed to fully understand their regulatory roles and capitalise on their therapeutic potential for improved management of mood disorders.

## AUTHOR CONTRIBUTIONS

Yogesh Dwivedi conceptualised the manuscript. Bhaskar Roy, Anuj K. Verma, Yu Funahashi, and Yogesh Dwivedi co‐wrote the manuscript. Yogesh Dwivedi finalised the manuscript.

## CONFLICT OF INTEREST STATEMENT

The authors declare no conflicts of interest.

### ETHICS STATEMENT

Not applicable.

## Data Availability

Data sharing is not applicable to this article as no new data were created or analysed in this study.
